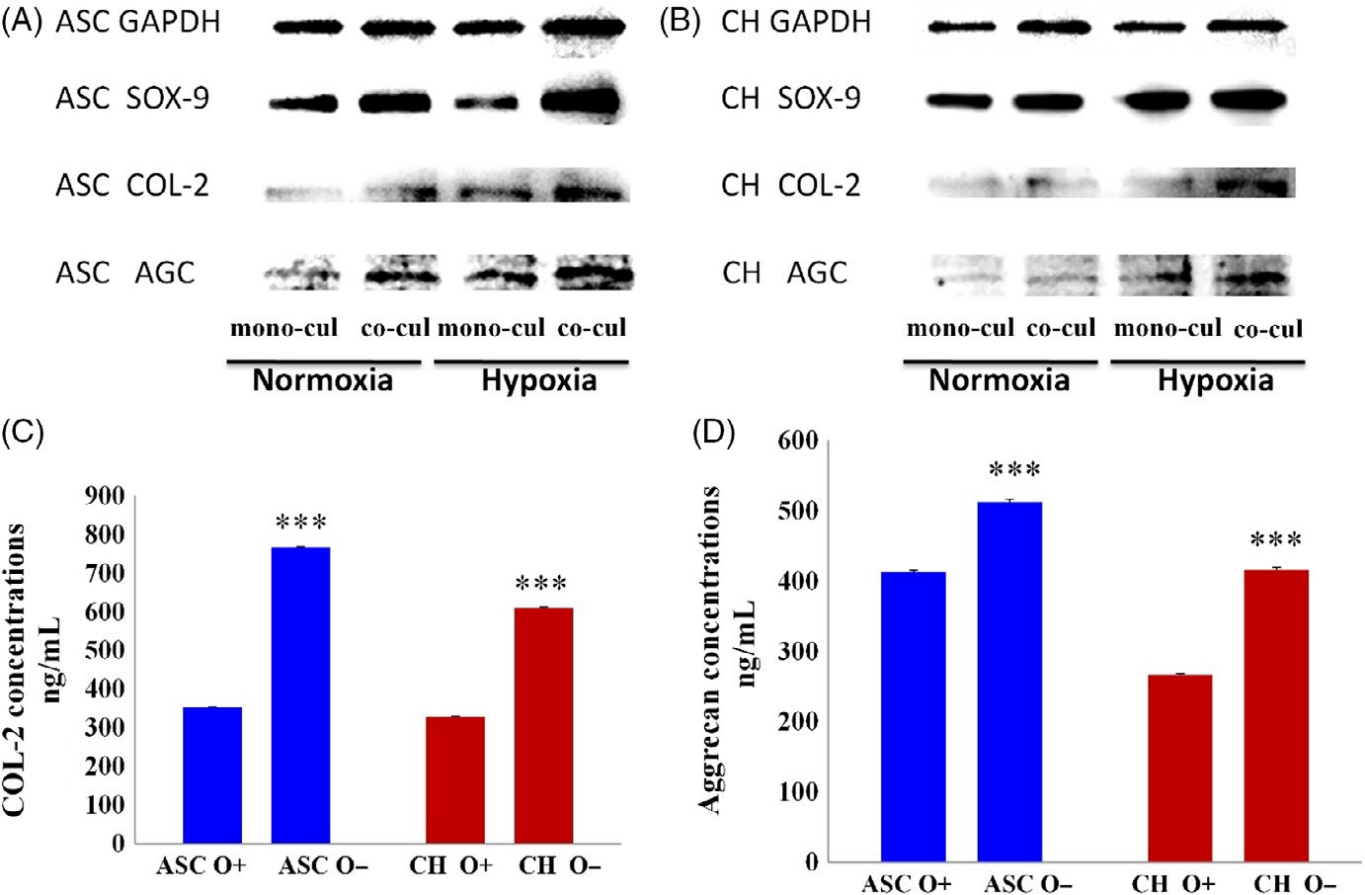# Corrigendum

**DOI:** 10.1111/cpr.12690

**Published:** 2019-11-21

*Cell Prolif*., 2016, **49**, 341–351

**Effects of low oxygen tension on gene profile of soluble growth factors in co‐cultured adipose‐derived stromal cells and chondrocytes**

Sirong Shi, Jing Xie, Juan Zhong, Shiyu Lin, Tao Zhang, Ke Sun, Na Fu, Xiaoru Shao and Yunfeng Lin

State Key Laboratory of Oral Diseases, West China Hospital of Stomatology, Sichuan University, Chengdu 610041, China

Received 22 October 2015; revision accepted 28 March 2016

The authors would like to draw the reader's attention to an error in Figure 3a (on page 346) in the above article: The Western blotting (WB) band of AGC in ASC was same as the WB band of AGC in CH. We have corrected it.

**Table:** 

Where	Wrong	Correction
In Figure 3A, the WB band of AGC in ASC was same with the WB band of AGC in CH.	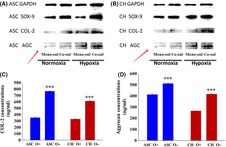	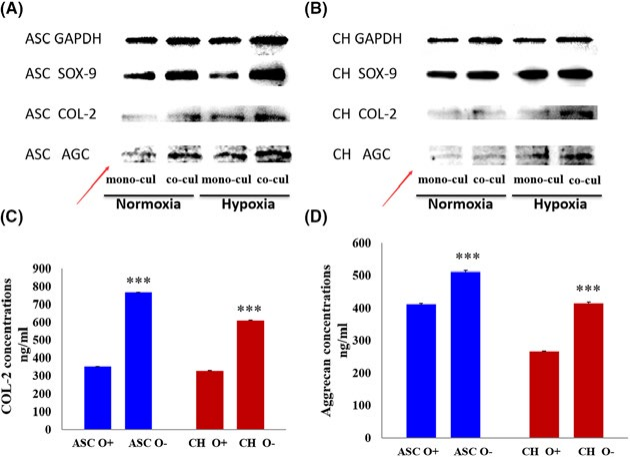

The revised Figure 3 is shown below.